# Humans with latent toxoplasmosis display altered reward modulation of cognitive control

**DOI:** 10.1038/s41598-017-10926-6

**Published:** 2017-08-31

**Authors:** Ann-Kathrin Stock, Danica Dajkic, Hedda Luise Köhling, Evelyn Heintschel von Heinegg, Melanie Fiedler, Christian Beste

**Affiliations:** 1 Cognitive Neurophysiology, Department of Child and Adolescent Psychiatry, Faculty of Medicine of the TU Dresden, Schubertstr. 42, 01307 Dresden, Germany; 2 Institute of Medical Microbiology, University Hospital Essen, University of Duisburg-Essen, Virchowstr. 179, 45147 Essen, Germany; 3 Institute of Virology, University Hospital, University of Duisburg-Essen, Virchowstr. 179, 45147 Essen, Germany; 4 Experimental Neurobiology, National Institute of Mental Health, Klecany, Czech Republic

## Abstract

Latent infection with *Toxoplasma gondii* has repeatedly been shown to be associated with behavioral changes that are commonly attributed to a presumed increase in dopaminergic signaling. Yet, virtually nothing is known about its effects on dopamine-driven reward processing. We therefore assessed behavior and event-related potentials in individuals with vs. without latent toxoplasmosis performing a rewarded control task. The data show that otherwise healthy young adults with latent toxoplasmosis show a greatly diminished response to monetary rewards as compared to their non-infected counterparts. While this selective effect eliminated a toxoplasmosis-induced speed advantage previously observed for non-rewarded behavior, Toxo-positive subjects could still be demonstrated to be superior to Toxo-negative subjects with respect to response accuracy. Event-related potential (ERP) and source localization analyses revealed that this advantage during rewarded behavior was based on increased allocation of processing resources reflected by larger visual late positive component (LPC) amplitudes and associated activity changes in the right temporo-parietal junction (BA40) and left auditory cortex (BA41). Taken together, individuals with latent toxoplasmosis show superior behavioral performance in challenging cognitive control situations but may at the same time have a reduced sensitivity towards motivational effects of rewards, which might be explained by the presumed increase in dopamine.

## Introduction

Latent toxoplasmosis is a very common chronic parasitic infection that is caused by the protozoan parasite *Toxoplasma gondii* (*T. gondii*) and has an estimated prevalence of 30–70% among human populations worldwide^1–4^. While most latently infected individuals have traditionally been considered to be clinically asymptomatic^5, 6^, there is now mounting evidence that latent infection causes several behavioral changes even in immunocompetent individuals^2, 7–10^. Yet, some important functions like reward-related modulations of behavior and cognitive control have not yet been investigated, even though *T. gondii* is likely to have profound effects on how rewards modulate our behavior:

*T. gondii* infects a wide range of warm-blooded animals including humans as secondary hosts^10, 11^ and one of the main factors contributing to its worldwide propagation is the ability to effectively alter the behavior of these secondary hosts (manipulation hypothesis)^10, 12, 13^. *T. gondii* has been shown to induce behavioral changes in rodents (increased exploratory behavior, decreased neophobia, and loss of fear of cats) which increase the risk of predation by a feline and thus ultimately benefit the parasite by completing its reproductive cycle^4, 14, 15^. One of the presumed main factors driving these behavioral changes is a potentially strong increase in dopaminergic signaling during both acute and latent infection^6, 8, 16, 17^. Direct histological evidence of this in otherwise healthy humans is still very scarce but this is at least partly due to the fact that practical and ethical reasons forbid the use of many of the techniques used for rodents to be applied in humans. Partly compensating for this, several studies on patients with schizophrenia, which is linked to pathologically increased dopaminergic signaling, have provided compelling evidence that changes in dopamine synthesis and release provide a direct link between toxoplasmosis and associated pathological changes in both brain and behavior^8, 17–19^. Based on this and other lines of evidence, it is deemed very likely that all infected secondary hosts show an up-regulation of dopamine and display associated behavioral changes^10^, although the number and location of cysts might play a big role for the quality and extent of the observed changes^8, 20^. Yet still, there is a broad consensus that based thereon, *T. gondii* should also manipulate human behavior^2, 10^. While a healthy immune system can keep the infection in check, it cannot effectively eliminate the parasite from the brain because neurons lack the MHC class I as well as specific intracellular mechanisms to inhibit parasite growth^6, 21, 22^. As a consequence, latent toxoplasmosis usually persist lifelong^5, 11, 23, 24^ and may thus potentially increase dopaminergic signaling and associated behavior irrespective of when/how long ago the host acquired the infection.

As dopamine plays a key role in many different cognitive functions, latent toxoplasmosis may have multiple effects on infected individuals. Two cognitive domains which have repeatedly been shown to be modulated by dopamine are cognitive control and reward/motivation^25^. But while several recent studies have shown toxoplasmosis-associated changes in executive functions such as inhibition, task switching, and working memory^7, 9, 26^, virtually nothing is known about whether or how latent toxoplasmosis alters the effects of rewards on behavior in otherwise healthy individuals, even though rewards have been shown to modulate the before-mentioned cognitive processes^27–29^. In rodents, *T. gondii* infection has been associated with a greater tolerance of reward forfeiture^30^, more risk-taking/less risk aversion^30, 31^, and an increased delay aversion for rewards^32^. In humans, latent toxoplasmosis has so far only been associated with increased sensation seeking^33^ and addiction^34^ but it has not been investigated at all whether or how *T. gondii* alters the behavioral modulation by reward. The presumed increase in dopaminergic signaling induced by *T. gondii* could amplify the effects of reward because increases in the dopamine response to reward have been shown to reflect its subjective value as well as formal economic utility^35^. If this was the case, latently infected individuals should respond more strongly to rewards and/or be inherently more motivated. However, the brains of latently infected individuals might also have homeostatically adjusted to the permanently elevated dopaminergic signaling and thus become less sensitive towards dopaminergic reward signals. Against this background, the supposed increase in sensation seeking^33^ could indicate that latently infected individuals require more stimulation to feel rewarded. In this case, they should respond less to rewards.

To investigate this matter and test whether one of the above-mentioned hypotheses holds true, we asked latently infected and matched non-infected young healthy adults to perform a rewarded stop-change task^36–38^ which had previously been shown to dissociate the behavior and associated neurophysiological correlates of Toxo-negative and –positive individuals in the absence of rewards (i.e. better behavioral performance/faster responses and smaller P3 amplitudes in Toxo-positive individuals^9^). To analyze the potential effect of rewards on task performance measures, we compared the behavioral data obtained from rewarded Toxo-positive and –negative groups with each other and furthermore separately compared both Toxo groups to the data obtained in a previous, non-rewarded study using the same experimental paradigm^9^ (Please note that neurophysiologic data could not be directly compared between studies as the current and the previous studies were conducted in two different labs with different EEG hardware).

To assess which cognitive sub-processes differ between rewarded Toxo-positive and –negative individuals, we quantified several event-related potentials (ERPs): Firstly, we quantified the P3, which is generally thought to link perception and action/reflect stimulus-response matching^39^. The P3 at fronto-central electrodes had previously been shown to reflect behavioral performance differences based on task goal processing strategies and to furthermore be modulated by dopamine in the context of our experimental paradigm^38, 40^. Moreover, the P3 at parietal sites had previously been associated with improved action control/faster responses in non-rewarded Toxo-positive individuals performing this task^9^. Additionally, we quantified the visual P1 and N1 ERPs to assess potential differences in early attentional stimulus processing^41–43^ as well as the visual late positive component (LPC). While none of our previous studies indicated a link between dopaminergic changes or toxoplasmosis and modulations of P1 and N1 amplitudes, they have been shown to be modulated by other factors like GABAergic signaling (which may be influenced by dopamine^44^) and were thus quantified to provide a more complete picture. While the LPC has not yet been shown to be altered by dopamine or latent toxoplasmosis, it has been demonstrated to be modulated by the intrinsic motivational significance of visual stimuli, as defined by increased autonomic responses and reports of greater affective arousal^45, 46^. Based thereon, it has been argued that the LPC might indicate “a selective processing of emotional stimuli, reflecting the activation of motivational systems in the brain”^45^. Given that the LPC has been reported to reflect motivated attention towards task-relevant information, including the prioritization of visual information related to behavioral performance^47^, we analyzed this ERP component.

**Figure 1:**
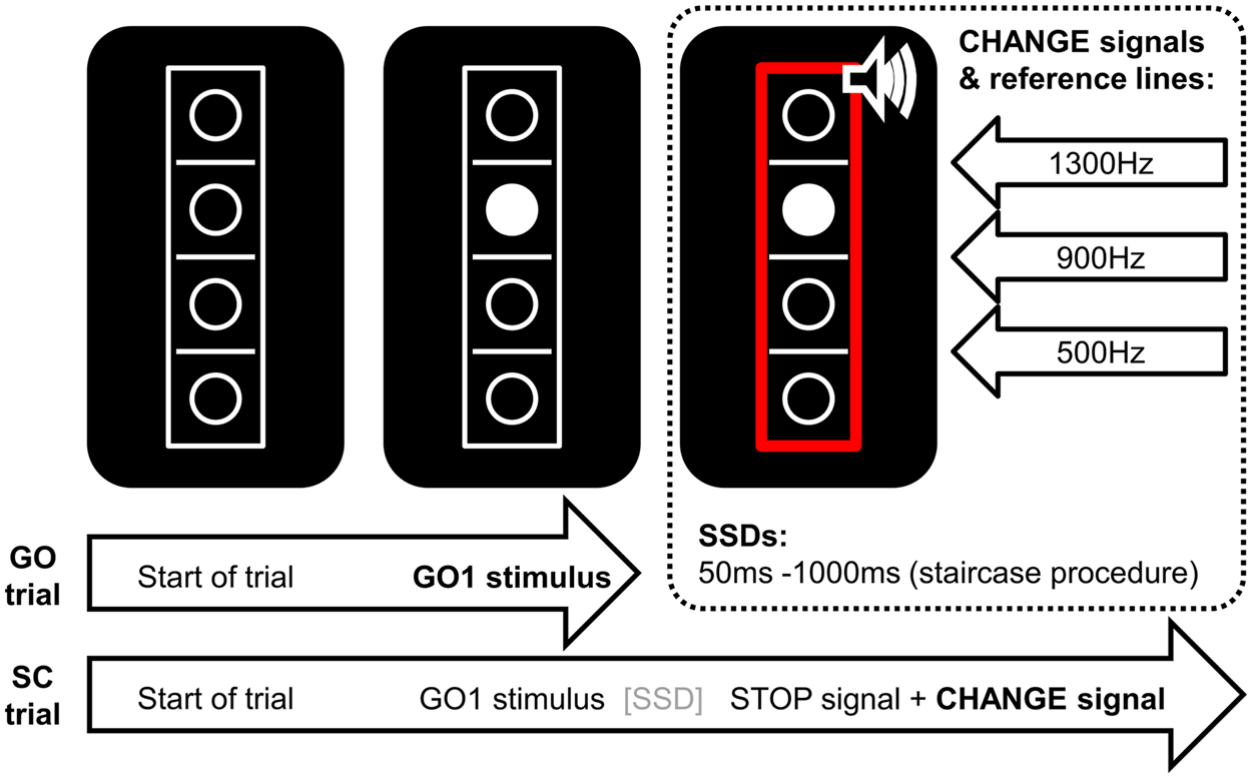
Illustration of the Stop-Change Task. Simple GO trials end after the first response to the GO stimulus (bold). In contrast, Stop-Change (SC) trials end after the first response to the CHANGE signal (bold). The stop-signal delay (SSD) between the onset of the GO stimulus and the STOP signal was adjusted using a staircase procedure described in the methods section. STOP and CHANGE stimuli were always presented simultaneously. As indicated in the upper right corner, the three CHANGE stimuli were associated with one of the three reference lines (see methods section for details).

Taken together, we investigated whether latent infection with the protozoan parasite *T. gondii*, which is known to increase dopamine synthesis and release in rodents and assumed to do the same in humans, alters the effects of rewards on behavior. For this purpose, we asked n = 14 young healthy latently infected (Toxo-positive) individuals and n = 14 matched non-infected (Toxo-negative) counterparts to perform a rewarded Stop-Change Task^48^ (for details, please refer to Fig. 1 and the methods section) that had previously demonstrated behavioral differences between infected and uninfected healthy young adults^9^. Both rewarded groups were compared to each other and to the behavioral data obtained in a previous study investigating the effect of latent toxoplasmosis with an identical, but unrewarded paradigm. ERPs were not compared across studies because the current and previous samples had been assessed in two differently equipped EEG labs.

## Results

### Behavioral results

We first analyzed the 576 GO trials, in which the participants were required to perform a simple right hand response (either index or middle finger button press) to a centrally presented visual stimulus (for details, please see methods section). The univariate ANOVAs for accuracy and hit RTs of GO trials revealed no significant differences between the two groups (all F(1,26) ≤ 1.933; p ≥ 0.176).

We then analyzed the 144 Stop-Change (SC) trials in which a right hand response to an initially presented GO stimulus had to be inhibited/aborted upon the presentation of a visual STOP stimulus while an alternative response to a simultaneously presented auditive CHANGE stimulus had to be carried out with the left hand (for details, please see methods section). The univariate ANOVA of SC trial accuracy yielded a main effect of group (F(1,26) = 5.445; p = 0.028; η²_P_ = 0.173) with Toxo-negative subject showing significantly lower accuracy values (60.49% ± 11.22) than Toxo-positive subjects (68.43% ± 6.01). The analysis of SC trial hit RTs did not yield a significant group difference (F = 1.483; p = 0.234). This lack of group differences stands in a striking contrast to a previous study where we had applied the same experimental paradigm (minus the reward manipulation used in this study) to examine the difference between Toxo-negative and Toxo-positive healthy young adults^9^. In that previous unrewarded study, the SC hit RTs of Toxo-positive subjects were significantly faster (949.78 ms ± 171.48) than those of Toxo-negative subjects (1121.47 ms ± 196.64). As the previous and current sample were recruited using the same inclusion and exclusion criteria and furthermore did not differ with respect to age (t = −0.039; p = 0.969), we compared the SC hit RTs from this study to the mean hit RT values obtained in the unrewarded (and unrelated) sample of our previous study (i.e. we compared the Toxo-negative group of this study to the mean value of the Toxo-negative group of the previous unrewarded study and likewise, the Toxo-positive group of the current study to the mean value of the Toxo-positive group of the previous unrewarded study). The tests revealed that with a mean difference of −288 ms, the rewarded Toxo-negative subjects responded significantly faster than the non-rewarded Toxo-negative subjects assessed in the previous, unrewarded study (t = −4.739; p < 0.001). In contrast to this, the rewarded Toxo-positive subjects did not differ from the unrewarded Toxo-positive subjects assessed in the previous, unrewarded study (t = −0.561; p = 0.584; mean difference: −25 ms).

Taken together, the behavioral analyses showed that rewarded Toxo-positive subjects had a higher accuracy rate in the SC condition than rewarded Toxo-negative subjects. Unlike what was found in previous studies, Toxo-negative and –positive subjects did not differ with respect to response times. Yet, a direct comparison with “baseline” data obtained in a previous, unrewarded study^9^ suggests that in case of complex stimulus input which requires both inhibition cognitive flexibility, rewards speed up responses in Toxo-negative subjects, but not in Toxo-positive subjects.

### Neurophysiological results

Given that only the SC condition showed group differences, we focused our neurophysiological analyses of this condition and omitted the simple GO condition that had not shown behavioral group differences.

The analyses of the visual P1 and N1 amplitudes at electrodes P7 and P8 did not reveal any significant effects (all F ≤ 1.977; p ≥ 0.172).

**Figure 2:**
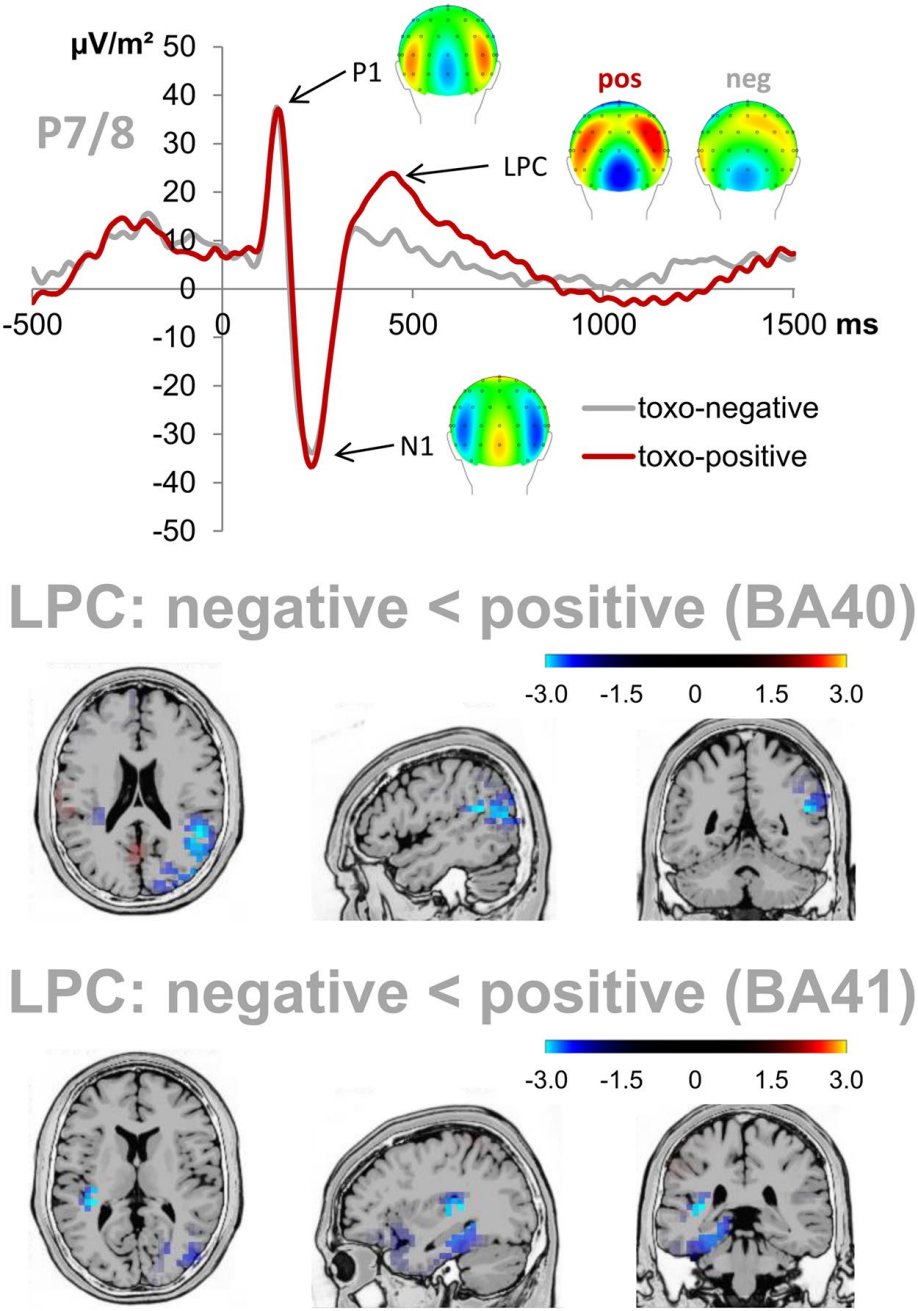
Attentional ERPs. The top graph depicts the visual P1, N1, and LPC event-related potentials at electrodes P7 and P8 (averaged) in the SC condition. Time point zero denotes the onset of the combined STOP + CHANGE stimuli. While the mean amplitudes of the P1 and N1 did not differ between groups (all p ≥ 0.172), LPC amplitudes were significantly larger in the Toxo-positive group than in the Toxo-negative group (p = 0.001). sLORETA source localization of this LPC group difference revealed a larger activation in the Toxo-positive group for both the right BA40 (middle row) and the left BA41 in the (bottom row).

The amplitudes of the visual LPC at electrodes P7 and P8 showed a main effect of group (F(1,26) = 12.604; p = 0.001; η²_P_ = 0.326) with larger amplitudes in the Toxo-positive group (24.460 µV/m² ± 2.651) than in the Toxo-negative group (11.152 µV/m² ± 2.651) which is plotted in Fig. 2. sLORETA source localization of this group difference within the LPC quantification time window from 420 to 440 ms revealed a larger activation in the Toxo-positive group for both the right BA40 and the left BA41 in the (see Fig. 2). The main effect of electrode as well as the interaction of electrode and group were non-significant (all F ≤ 4.176; p = 0.051).

**Figure 3:**
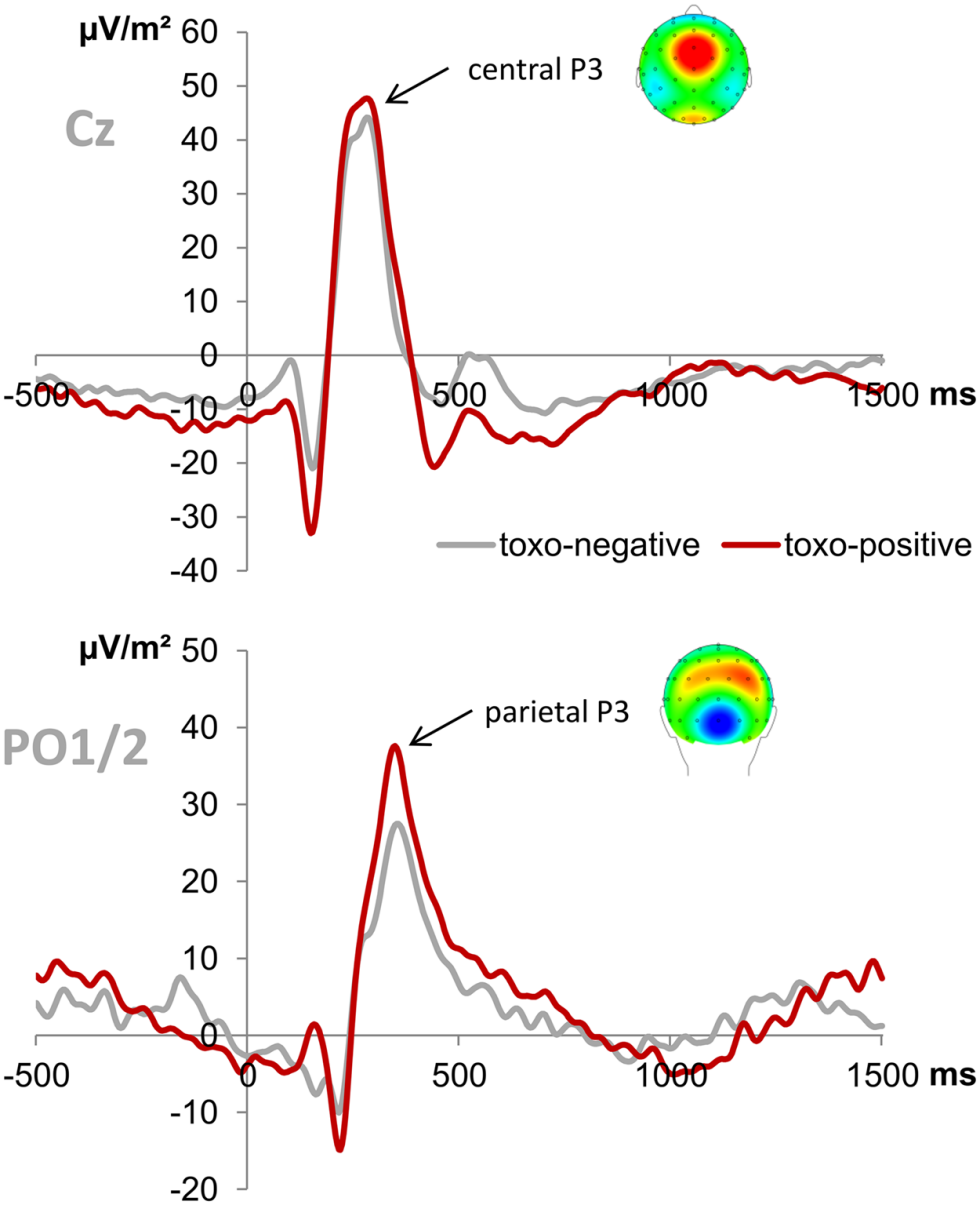
P3 ERPs. The top graph denotes the central P3 peak at electrode Cz in the SC condition. The bottom graph depicts the parietal P3 peak at electrodes PO1 and PO2 (averaged) in the Stop-Change (SC) condition. Time point zero denotes the onset of the combined STOP + CHANGE stimuli. None of the group differences reached significance (all p ≥ 0.109).

The P3 ERP at both quantification sites is plotted in Fig. 3. The P3 amplitude quantified at electrode Cz did not reveal a significant group difference (F(1,26) = 0.313; p = 0.581). Also, the P3 amplitude quantified at electrodes PO1 and PO2 did not reveal any significant effects of group, electrode, or their interaction (all F ≤ 2.754; p ≥ 0.109).

### Statistical Power

As the sample only contained 14 subjects in both groups, we conducted additional power analyses to determine the achieved power. For the main effect of group on accuracy and LPC amplitudes we hence conducted two separate analyses using the G*Power software package^49^. First, the obtained η²_P_ were converted to f-values (accuracy: η²_P_ = 0.173 was converted to f = 0.4575728; LPC: η²_P_ = 0.326 was converted to f = 0.6954707) and then entered in the achieved power calculation for one-way ANOVAs (each with an alpha error probability of 0.05, a total sample size of n = 28 and 2 groups). This yielded a power of 64.48% for the SC accuracy analysis and a power of 94.31% for the LPC analysis. This shows that while the behavioral analysis would have benefited from a larger sample, the neurophysiological analysis was sufficiently powered and can thus be regarded as reliable.

### Exposure to (other) infectious agents

In order to exclude the possibility that the observed Toxo-related behavioral results were moderated by general differences in the exposure to common viral or bacterial infections, that may also be responsible for undetected unspecific neurologic disorders^50^, we conducted additional analyses to assess IgG antibodies to common infectious agents, which would not be expected to alter dopamine in the brain (i.e. Human Cytomegalovirus, Herpes simplex virus 1 and 2, Epstein-Barr virus, Respiratory Syncytial virus, Adenovirus, Parainfluenza virus Type 1, 2 and 3, *Chlamydia pneumoniae* and *Mycoplasma pneumoniae*). The selection of these infectious agents was based on the assumption that they are predominantly acquired in childhood, as the subjects were mainly students aged 18 to 30, where endemic infection and consecutive seroprevalence are expected to be at a high level in Germany. Also, there is no vaccination recommendation of the German Standing Committee on Vaccination (STIKO) against the tested infectious agents.

The results of our analyses revealed common prevalence values, none of which significantly differed between the *Toxo-positive* and *Toxo-negative* groups (all p ≥ 0.246; see supplementary material for details). Also, none of the additionally investigated infections caused a significant behavioral difference, as observed for latent toxoplasmosis (all p ≥ 0.156; see supplementary material for details).

## Discussion

*T. gondii* has been shown to manipulate the behavior of its secondary hosts, which is commonly assumed to be at least partly driven by an unspecific increase in dopamine synthesis and –release^2, 16, 17^. Based thereon, we investigated whether latent toxoplasmosis also affects the reward-related modulation of cognitive control, as the effects of rewards heavily depend on dopaminergic signals^35^. To elucidate this question, we compared behavioral and neurophysiological measures of Toxo-positive and–negative healthy young adults performing a rewarded stop-change task, that had previously shown superior performance in Toxo-positive subjects^9^.

At the behavioral level, we found that in the SC condition, rewarded Toxo-positive subjects responded with a higher accuracy than rewarded Toxo-negative subjects. Yet, a comparison with data obtained from a previous non-rewarded study using the same paradigm^9^ revealed that there was no RT difference between groups in rewarded participants whereas non-rewarded Toxo-positive participants had previously shown markedly faster responses than Toxo-negative ones^9^. Further statistical comparisons of the data obtained in the current vs. previous study revealed that rewarding participants seems to significantly speed up correct responses of Toxo-negative subjects, but not those of Toxo-positive subjects. Taken together, these findings suggest that both in rewarded and non-rewarded experimental setups, latent toxoplasmosis (and the assumed increase in dopaminergic signaling) seems to improve behavioral performance in case of very challenging simultaneous stimulus input. Given that only the responses of Toxo-negative subjects seem to be speeded up by rewards, it can however also be stated that the effects of rewards on behavioral performance were greatly diminished in Toxo-positive individuals. This finding supports the second hypothesis outlined in the introduction, namely that Toxo-positive individuals respond less to rewards as they might have become less sensitive towards modulations of the dopaminergic reward signal due to the presumed constantly elevated dopaminergic signaling. Given that rewards had improved the RTs of Toxo-negative subjects to the performance level of Toxo-positive subjects, it can however not be stated that rewarded Toxo-positive individuals are generally less motivated than rewarded Toxo-negative individuals. Of note, this behavioral effect of toxoplasmosis on reward processing could not be explained by the exposure to (other) infectious agents that may also be responsible for undetected unspecific neurologic disorders^50^ (see supplementary material for details). While we definitively require further studies to either substantiate or disprove this claim, it could be possible that the assumed chronic increase in dopaminergic signaling of Toxo-positive individuals^8, 16, 17^ chronically enhances their dopamine levels, thus mimicking the effects of reward found in non-affected individuals. Alternatively, it would however also be plausible that the presumed elevated dopamine levels only enable better cognitive control in Toxo-positive individuals but do not permanently increase motivation itself.

On the neurophysiologic level, we found that the higher SC accuracy rates of the Toxo-positive group were reflected by larger visual LPC amplitudes at parietal/occipital electrodes (P7/8). The LPC reflects motivated attention towards task-relevant information including the prioritization of visual information related to behavioral performance^47^. It has furthermore been associated with the allocation of attentional resources to changes^51^ as well as updating and perceptual reversal^52^, all of which are important to successfully perform the switch required in the SC condition. The fact that a larger LPC has also been associated with both successful inhibition and accuracy in a matching task^53^ nicely matches the finding of higher accuracy in the Toxo-positive group as accuracy is strongly dependent on successful inhibition in the SC condition (due to the applied staircase procedure; please compare methods section). With regard to reward, the LPC has been shown to be modulated by the intrinsic motivational significance of visual stimuli, as defined by increased autonomic responses and reports of greater affective arousal^45, 46^. Based thereon, it has been argued that the LPC might reflect “the activation of motivational systems in the brain”^45^. Trying to clarify the underlying mechanisms, Furley *et al*.^47^ have proposed that „higher-order neural hubs that code affective information or govern the distribution of attentional resources provide top-down information that feeds, in a reentrant fashion, into structures coding basic perceptual features” so that the visual representation of motivationally relevant stimuli is enhanced. It hence seems that accuracy differences between the two groups originate in how much cognitive resources are allocated to detecting and processing the task-relevant STOP + CHANGE stimulus. This was further supported by the findings of the sLORETA which revealed that the larger LPC amplitudes of the Toxo-positive group were based on larger activation within the right BA40 as well as the left BA41. This also matches previous reports stating that changes in LPC amplitudes originate in networks coding both sensory and affective features^47^. BA 40 is part of the inferior parietal lobule/temporo-parietal junction (TPJ) and has been associated with the top-down guided allocation of (visual) spatial attention^54–56^. It has furthermore been shown to play an important role in both the ventral attention network and the fronto-parietal control network^55^ and to be relevant for dual task performance as it sustains executive control^56, 57^. Lastly, especially the right TPJ has been shown to play a critical role for the detection of novel and behaviorally relevant stimuli, attentional reorienting, target detection, and processing of competing stimuli^54^. Also, the TPJ is concerned with post-perceptual processes involved in contextual updating and adjustments of top-down expectations^58^. All taken together, these findings on TPJ function nicely match the claim that the LPC could reflect perceptual reversal and its post-perceptual appraisal as well as task-relevant working memory updating^52, 59^. Based thereon, it can be inferred that changes in the activity of the right BA40 in our study should reflect the detection and reorienting to the visual STOP + CHANGE stimulus and probably also contextual working memory updating. In contrast to this, BA41 is part of the primary auditory cortex which is central to the accurate perception of pitch/tone frequency^60, 61^. Given that the relevant CHANGE signal information was coded by the pitch, it can be inferred that activation differences in the left BA41 should reflect better processing of the incoming task-relevant CHANGE stimulus information. Successful inhibition has been suggested to rely on the adjustment of attentional settings to optimize stimulus detection and evaluation^62–64^, and the LPC has been implied in both inhibition^53^ and attentional reorientation towards task-relevant stimuli^47^. Based thereon, it can be stated that the higher accuracy of rewarded Toxo-positive subjects seems to be based on the an increased allocation of cognitive resources to processing task-relevant features of the visual STOP stimulus (right BA 40) as well as the auditory CHANGE stimulus (left BA41).

In contrast to the LPC, the P1 and N1 ERPs, which reflect early attentional processing of visual stimuli^41^, did not reflect group differences. Together with the fact that none of our previous studies found these two ERPs to be modulated by dopamine in the stop-change task^38, 40^, this supports the notion that toxoplasmosis primarily affects top-down cognitive control processes^9^, but not attentional processes. The lack of differences in both the central and parietal P3 ERPs, which reflect stimulus-response matching^39^, was however unexpected because these processes have previously been shown to be modulated by dopamine in this experimental paradigm^38, 40^. Furthermore, the parietal P3 had previously been associated with improved action control/faster responses in non-rewarded Toxo-positive individuals performing this task^9^. In both cases (i.e. central and parietal P3 amplitudes), smaller amplitudes had previously been associated with better behavioral performance. This lack of expected effects could however be explained by the nature of group differences found in this study: The association of P3 amplitude differences with behavioral performance differences was always based on hit RTs (i.e. faster responses in one of the respective investigated groups), but the effects of our current study were based on accuracy, i.e. a different behavioral measure. Given the reduced responsivity of Toxo-positive individuals towards rewards, one might speculate whether the stronger reward response of Toxo-negative subjects might have increased their levels of released dopamine to equal the presumed chronically elevated dopamine release in Toxo-positive individuals, thus possibly ‘evening out’ some of the previously observed effects (i.e. hit RT and parietal P3 differences).

However, the current study has a few limitations which shall also be discussed: Latent toxoplasmosis is a lifelong infection so that prevalence increases with age. Due to this reason, our initial screening only yielded 14 Toxo-positive individuals. While this yielded a sufficient power for the ERP analysis as determined post-hoc, the behavioral analyses would surely have benefitted from additional subjects. Our findings should therefore be substantiated by further studies with ideally larger sample sizes. -Also because a replication in another, independent sample would substantiate the claim that the differences we found are truly specific to latent toxoplasmosis. Furthermore, studies with performance-based rewards would be a useful follow-up to our current study, which was based on sham-rewards that only seemed to be based on performance. The reason for this is that our previous study had shown Toxo-induced performance differences^9^, which might have confounded the effects of reward (for elaboration on this, please see methods section). Furthermore, we recruited all subjects from a population of Caucasian university students of Western European descent (which ensured a quite homogenous group with respect to socioeconomic and genetic factors) that all stated to be healthy and free of any psychiatric or neurologic disease. At least the latter may have excluded functionally impaired individuals with latent toxoplasmosis and may thus have potentially induced a selection bias. Lastly, we did not directly assess changes in dopaminergic signaling, but only its effects on rewarded behavior. It is an undisputed fact that reward effects are mainly mediated by dopamine^25^ and it has also been well-established that latent toxoplasmosis enhances dopaminergic signaling in rodents^6, 8, 16, 17^. While the direct histological proof of enhanced dopamine synthesis and release in humans still needs to be established, it is deemed very likely by leading experts in the field^6, 8, 17, 18^ so that we deem it likely that the reported effects may be based on *T. gondii*-induced changes in dopaminergic signaling. Yet still, *T. gondii* has several direct and indirect effects on the brains of its secondary host (for review, please refer to Parlog *et al*.^6^) so that we cannot entirely exclude the possibility that other factors such as neuroplasticity or apoptosis may have contributed to our findings.

Taken together, individuals with latent toxoplasmosis showed a greatly diminished the response to monetary rewards and its effect on cognitive control processes as compared to non-infected individuals. This does however not imply impaired behavioral performance as the Toxo-positive subjects could still be demonstrated to be superior to Toxo-negative subjects with respect to response accuracy in a cognitively challenging multitasking condition. Specifically, the better performance of rewarded Toxo-positive individuals was based on the increased allocation of processing resources serving the detection of and reorienting to task-relevant visual and auditory stimuli as reflected by larger LPC amplitudes and higher BA40 and BA41 activity. This suggests that the presumed chronic increase in dopaminergic signaling in latent toxoplasmosis may boost behavioral performance in challenging cognitive control situations but may at the same time reduce the sensitivity towards motivational effects of rewards.

## Methods

### Sample

We screened a sample of n = 131 young healthy subjects for latent toxoplasmosis (for details, please refer to the text section *“Assessment of Toxoplasma gondii antibodies”*). All screened individuals were students of the TU Dresden who had been recruited via online ads and flyers. Inclusion criteria were age (18-30 years) and right-handedness. Exclusion criteria were any psychiatric or neurological diseases, any current or regular medication (with the exception of oral contraceptives for females) as well as any impairments of hearing or vision. The initial screening sample yielded n = 14 (4 ♂, 10 ♀) Toxo-positive individuals which were invited to take participate in the study. An equally large Toxo-negative subsample n = 14 (4 ♂, 10 ♀) matched for sex and age (±2 years) was invited to participate in the study as the control group. The status of latent toxoplasmosis was reassessed based on blood samples drawn on the day of the experiment to exclude potential mix-ups or new infections that might have occurred in between the initial screening and the day of the experiment. All data was collected in a double-blind protocol so that at the time of study participation, neither the experimenter nor the subjects knew who had latent toxoplasmosis and who did not. The characteristics and comparison of both samples are given in Table 1. All participants stated to be right-handed and had normal or corrected-to-normal vision and hearing. None the participants reported any history of psychiatric or neurologic disease, nor were any of the participants on regular or current medication (with the exception of oral contraceptives). Prior to the experiment, each participant gave written informed consent and received 50 € after taking part in the study. The study was approved by the ethics committee of the Faculty of Medicine of the TU Dresden and subjects were treated in accordance with the declaration of Helsinki.

**Table 1:** The table sums up the sociodemographic characteristics of the two groups.

	toxo-negative	toxo-positive	p-value of independent samples t-test
age in years	23.93 ± 2.95	25.07 ± 3.08	0.325
height in cm	170.71 ± 8.89	171.29 ± 9,47	0.871
weight in kg	60.25 ± 8.67	63.50 ± 12.48	0.431
cigarettes smoked per day	0.86 ± 2.66	0.27 ± 1.07	0.462
standard units of alcohol per week	2.79 ± 3.94	2.21 ± 2.45	0.649
BDI score	3.29 ± 3.91	2.71 ± 2.58	0.652

### Task

The task is illustrated in Fig. 1. Importantly, the task was identical to the one used in a previous study investigating the effects of toxoplasmosis on behaviour without reward^9^.

Participants were seated in a dimly lit and sound-attenuated room. Stimuli were presented on a 17′′ CRT monitor and via headphones with the help of Presentation (v. 17.1, Neurobehavioral Systems, Inc.). Responses had to be given with the help of four buttons located on a regular keyboard.

The task was a modified version of the stop-change paradigm by Verbruggen *et al*.^48^. At the start of each trial, a rectangular frame (20 × 96 mm) containing four vertically aligned circular frames (8 mm diameter) and three horizontal reference lines (line thickness: 1 mm; width: 8 mm) separating the circles were presented on black background in the center of the screen. After 250 ms, one of the circles was filled with white colour, thus turning into the GO stimulus (target). In the GO trials (80% of trials), participants were instructed to indicate whether the target was located above or below the middle reference line. Responses were given by pressing the “K” key with the right middle finger (“above” judgment) or by pressing the “N” key with the right index finger (“below” judgment) on the keyboard. The stimuli remained on the screen until the participant responded. In case of responses longer than 1000 ms, the German word “Schneller!” (engl. “Faster!”) was presented above the box until the participant responded and thereby ended the trial. The remaining 20% of the trials were stop-change (SC) trials. Like GO trials, SC trials started with the presentation of the empty array followed by the target. After a variable stop signal delay (SSD), a STOP signal (a red rectangle replacing the white frame) was presented. Upon its appearance, participants were required to try to inhibit their right hand response to the GO stimulus. The SSD was initially set to 450 ms and adapted to the participants’ performance by means of a ‘staircase procedure, roughly yielding a 50% probability of successfully inhibited GO responses. The STOP signal was simultaneously presented with a CHANGE stimulus, which was a 100 ms sine tone (75 db SPL) presented via headphones. It could be either high (1300 Hz), medium (900 Hz) or low (500 Hz). Each of the tones coded for one of the reference lines (high tone = high line medium tone = medium line, low tone = low line) so that the CHANGE signal in SC trials “assigned” a new reference line. All three tones/reference lines were in effect equally often. The required CHANGE response had to be performed with the left hand. If the target was above the newly indicated reference line, the “S” key press (left middle finger) was required and if the target was located below the newly assigned reference line, a “C” key press (left index finger) was required. In case of response times (RTs) longer than 2000 ms, the German word “Schneller!” (translates to “Faster!”) was presented above the box until the participant responded to end the trial. After each SC trial, the SSD for the next SC trial was adjusted by the above-mentioned staircase algorithm^48^. In case of a correct response (defined as a correct inhibition of GO response and a correct CHANGE response), the SSD was prolonged by 50 ms, and otherwise shortened by 50 ms. Due to this adaptive procedure, subjects respond incorrectly or prematurely in roughly half of the SC trials. To keep the experiment within reasonable limits, SSDs were confined to the range from 50 to 1000 ms. Each trial was followed by an inter-trial interval of 900 ms. Participants were instructed to respond as fast and accurately as possible. The experiment consisted of 720 trials divided into 6 blocks. The trial order was pseudo-randomized. Furthermore, each participant completed an extensive training block prior to the experiment.

### Reward manipulation

To investigate whether rewards differentially affect behavior in Toxo-negative and Toxo-positive individuals, we rewarded all participants of this study. Since a previous, non-rewarded study evidenced that latent toxoplasmosis may cause performance improvements in at least one of the investigated conditions^9^, we however refrained from issuing performance-based rewards. Instead, we only told participants that rewards were based on performance, when they were actually fixed. This was done as follows:

Before the start of the task, the participants received the information that they would start out with a basic reimbursement of 40 €. In each of the six blocks, they could ostensibly earn up to 2 € based on a “complicated ratio of speed and accuracy that was automatically determined by the computer program”. They could however not lose any money. By pretending that the reward was based on an overly complicated algorithm, we kept the subjects from counting correct answers or the like to estimate or predetermine their reward within each block. This procedure was successful as all participants stated be convinced that the rewards had “clearly reflected” their behavioral performance when questioned by the experimenter at the end of the data collection.

**Figure 4:**
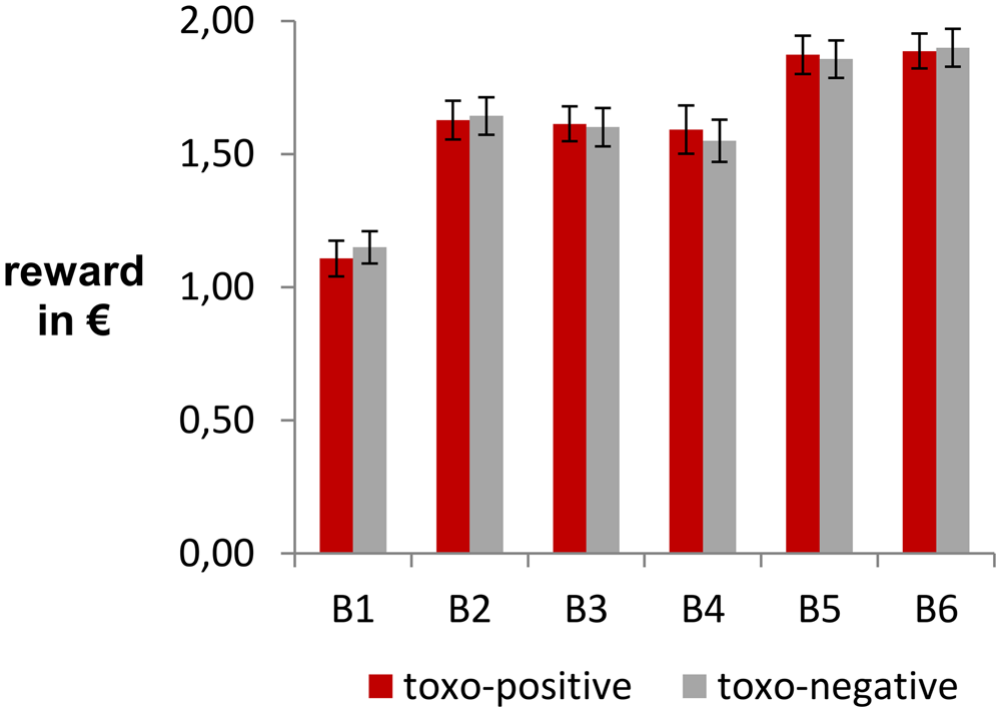
Reward manipulation. After each of the six experimental blocks, the participants received feedback on their ostensible performance-based rewards. The rewards were subject to slight random variations with each subject to conceal the fact that the rewards were not based on behavioral performance. After the completion of all six blocks, the individual rewards would always end up between 49,51 € and 49,99 €, which was then “generously” rounded up to 50 € by the experimenter. The bars indicate the mean rewards in each block and group while the error bars denote the respective standard deviation. Of note, the rewards of the two groups did not differ significantly in any of the blocks (all p ≥ 0.110).

After each block, the participants received feedback on their ostensible performance-based rewards (total amount earned as well as rewards earned in the previous block) in the middle of the screen. The block-wise rewards were designed to mimic some learning effects as a previous study using this paradigm indicated that performance improves over time^65^. To avoid a coincidental uncovering of our fixed rewards (e.g. by two participants accidentally knowing each other and comparing their individual rewards), the rewards were subject to slight random variations with each subject (see Fig. 4). After the completion of all six blocks, the individual rewards would however always end up between 49,51€ and 49,99€ which was then “generously” rounded up to 50 € by the experimenter. Of note, the rewards of the two groups did not differ significantly in any of the blocks (all p ≥ 0.110).

### EEG recording and ERP quantification

The EEG was recorded using a QuickAmp amplifier (Brain Products, Inc.) with 60 Ag–AgCl electrodes at standard equidistant scalp positions. The reference electrode was located at Fpz and all electrode impedances were set to <5 kΩ during recording. The data were recorded with a sampling rate of 500 Hz and later (offline) down-sampled to 256 Hz. Next, an IIR band- pass filter ranging from 0.5 to 20 Hz (with a slope of 48 db/oct each) and a notch at 50 Hz were applied. A manual inspection of the data was performed to remove technical artifacts and rare artifacts such as jaw clenching. Recurring artifacts caused by eye movements, heart rate etc. were removed by means of an independent component analysis (ICA) using the infomax algorithm as implemented in BrainVision Analyzer 2.1. Single trial segments were formed for correct SC trials only, as the GO condition did not reveal significant behavioral group differences (compare results section). To be rated as correct, the trials needed to contain correct responses to the CHANGE stimulus before the onset of the speedup-sign and no premature or incorrect button presses either before or after the CHANGE stimulus. The segments were stimulus-locked to the onset of the combined STOP/CHANGE stimulus, which was set to time point zero. Each segment started 1000 ms before the onset of the STOP/CHANGE stimulus and ended 1500 ms thereafter, resulting in an overall segment length of 4000 ms. An automated artefact rejection excluded all segments which met one or more of the following exclusion criteria: amplitudes below −100 μV or above 100 μV, value differences of more than 200 μV in a 200 ms interval, or value differences of less than 0.5 μV in a 100 ms interval. Next, a baseline correction was set to the time window from −900 to −700 ms to obtain a pre-stimulus baseline which was mostly unaffected by the GO stimulus presentation. Finally, the segments were averaged on the single-subject level so that ERPs could be quantified. At electrodes P7 and P8, we quantified the visual P1 ERPs by averaging the time window from 130 to 140 ms. At the same electrodes, the visual N1 amplitude was quantified from 220 to 235 ms and the LPC was quantified from 420 to 440 ms. Of note, the late positive component is an event-related potential which may sometimes be confused for a P3b^52, 66, 67^. It has consistently been described to start about 300 to 400 ms after stimulus onset^47, 52, 67–70^ and typically be maximal over posterior (parietal and occipital) electrodes for visual stimuli^47, 52, 53, 68, 70^, in our case over P7 and P8. The fronto-central and parietal P3 ERPs were quantified at electrode Cz from 255 to 285 ms and at electrodes PO1 and PO2 from 320 to 350 ms, respectively.

### ERP source localization

For source localization analyses, sLORETA (standardized low resolution brain electromagnetic tomography)^71^ was used, which provides a single solution to the inverse problem^71–73^. For sLORETA, the intracerebral volume is partitioned into 6239 voxels at 5 mm spatial resolution. Then, the standardized current density at each voxel is calculated in a realistic head based on the MNI152 template^74^. It has been mathematically proven that sLORETA provides reliable results without a localization bias^73^. Moreover, there is evidence from EEG/fMRI and neuronavigated EEG/TMS studies underlining the validity of the sources estimated using sLORETA^73, 75^. The voxel-based sLORETA images were compared across groups (Toxo-positive vs. Toxo-negative) using the sLORETA-built-in voxel-wise randomization tests with 2000 permutations, based on statistical nonparametric mapping (SnPM). Voxels with significant differences (p < 0.01, corrected for multiple comparisons) between contrasted conditions were located in the MNI-brain.

### Assessment of Toxoplasma gondii antibodies

The participants were classified as either Toxoplasma-positive or –negative based on the concentration of specific IgM and IgG anti-*Toxoplasma gondii* antibodies in their sera. IgG and IgM antibodies were detected in sera of candidates using a chemiluminescence immunoassay (LIAISON Toxo IgG II and IgM; DiaSorin, Saluggia, Italy) running on a LIAISON XL (DiaSorin, Saluggia, Italy), a fully automated chemiluminescence analyzer. Inactivated *Toxoplasma gondii* (RH strain) from sonicated and extracted trophozoites serve as test antigen. According to the instructions of the manufacturer, samples with concentrations of anti-*Toxoplasma gondii-*IgG antibodies greater than or equal 8.8 IE/mL were considered reactive for specific IgG-antibodies to *Toxoplasma gondii*. Samples with antibody concentrations less than 7.2 IE/mL were considered nonreactive for IgG. The diagnostic specificity of the IgG-test is 99.43% with a confidence level of 98.56–99.84% and the sensitivity is 100% with a 95%-confidence level of 98.45–100%. According to the instructions of the manufacturer, samples with concentrations of anti-*Toxoplasma gondii*-IgM antibodies greater than or equal 8 AU/mL were considered reactive for specific IgM-antibodies to *Toxoplasma gondii*. Samples with antibody concentrations less than 6 AU/mL were considered non-reactive for IgM. The diagnostic specificity of the IgM-test is 98.49% with a 95%-confidence level of 97.15–99.31% and the sensitivity is 100% with a 95%-confidence level of 97.45–100%. On average, the Toxo-positive group had an IgG of 59.44 IU/ml (SD = 39.52; range 17.4–174). Additionally, one of the subjects of the Toxo-positive group was tested positive for IgM antibodies (11.4 IU/ml with an avidity of 70.8%). Of note, none of the subjects included in this study were within the equivocal ranges.

### Assessment of antibodies to other infectious agents

IgG antibodies to other infectious agents could only be assessed in the reward group as we only had retained serum samples of this cohort. The details and results of these additional procedures are provided in the supplementary material.

### Statistics

Separate univariate ANOVAs were used to analyze each behavioral and ERP measure. However, only conditions which yielded significant group differences in the behavioral data were investigated during ERP analyses. All analyses used “group” (Toxo-positive vs. Toxo-negative) as a between-subjects factor and were separately conducted for the GO and SC conditions. Additionally, the within-subjects factor “electrode” was used wherever applicable. All reported values underwent Greenhouse-Geisser correction and post-hoc tests were Bonferroni-corrected, whenever necessary. For all descriptive statistics, the standard error of the mean (SEM) is given as a measure of variability.

## Electronic supplementary material

Additional serological analyses
