# Supplementary material for: Humans with latent toxoplasmosis display altered reward modulation of cognitive control
Source: Sci Rep. 2017 Aug 31;7:10170. doi: 10.1038/s41598-017-10926-6 (PMC5579228; doi:10.1038/s41598-017-10926-6)
Supplement: Supplementary file 1 — Additional serological analyses [file 41598_2017_10926_MOESM1_ESM.pdf]

# Supplement

| Title | Humans with latent toxoplasmosis display altered reward modulation of cognitive control |
|---|---|
| **Authors** | Stock AK, Dajkic D, Köhling HL, Heintschel von Heinegg E, Fiedler M, Beste C |
| **Journal** | Scientific Reports |

As explained in the main manuscript, we conducted additional analyses to assess IgG antibodies to common infectious agents (i.e. Human Cytomegalovirus (CMV), Herpes simplex virus (HSV) 1 and 2, Epstein-Barr virus (EBV), Respiratory Syncytial virus (RSV), Adenovirus, Parainfluenza virus Type 1, 2 and 3, *Chlamydia pneumoniae* and *Mycoplasma pneumoniae*) to exclude the possibility that the observed toxo-related behavioral results were moderated by a general difference in the exposure to common viral or bacterial infections [1]. The selection of these infectious agents was based on the assumption that they are predominantly acquired in childhood, as the subjects were mainly students aged 18 to 30, where endemic infection and consecutive seroprevalence are expected to be at a high level in Germany. Also, there is no vaccination recommendation of the German Standing Committee on Vaccination (STIKO) against the tested infectious agents.

## Results

### *Prevalence*

IgG antibodies against the three herpesviruses CMV, EBV and HSV were detected in 39.3 %, 74.1 % and 42.9 % of the subjects, respectively. This EBV seroprevalence is in concordance with rates of 68 to 78 % from students in Scotland [2], whereas other epidemiological studies found a slightly higher IgG antibody prevalence against CMV and HSV 1 (56 - 64 % and 85 - 89 %, respectively) [3,4]. In our cohort, every subject had positive IgG antibody levels against RSV and Parainfluenza, which has been described previously: almost 100 % of the adults worldwide exhibit RSV antibodies [5] and almost every child experienced an infection with Parainfluenza until the age of five [6]. At the age of 20 to 29, the IgG antibody prevalence against *Mycoplasma pneumoniae* is about 40 % according to a study in a healthy Finnish population [7]. Our measured seroprevalence of 32.1 % is lower. Tuuminen et al. [7] also reported a seroprevalence of IgG antibodies against *Chlamydia pneumoniae* at the same age of about 60 %. The measured prevalence in our group was lower with 32.1% for *C. pneumoniae* IgG antibodies. Antibodies against Adenovirus were found positive in 92.9 % of the subjects. This is in concordance with the study of Mast et al.[8] who reported that the seroprevalence of Adenovirus IgG in Europe was 93 %.

In short, these results suggest that rate of IgG antibodies against common viral and bacterial infections detected in our sample is comparable to the prevalence / antibody titers reported for healthy young adults in Germany and Europe.

### *Association between T. gondii and the other assessed infections*

The potential association between *T. gondii* and the other assessed infections was investigated with the help of separate Chi square tests of independence / association. There was no statistically significant association between latent *T. gondii* infections and CMV ($\chi 2(1) = 1.348$, $p = .246$) or between *T. gondii* and HSV ($\chi 2(1) < 0.001$, $p > .999$). As at least one of the expected cell frequencies was smaller than five for the other infections, Fisher's exact tests were run for them instead. Those indicated that neither EBV ($p = .385$), *Mycoplasma*

*pneumoniae* (p = .420), Adenovirus (p = .481), nor *Chlamydia pneumoniae* (p = .420) significantly differed between *Toxo-positive* and *Toxo-negative* study participants. RSV and Parainfluenza antibodies were not analyzed as all study participants had been tested positive.

*Behavioral effects of the other assessed infections*

As *T. gondii* had shown a significant effect on SC trial accuracy (see main document), we used separate Mann-Whitney-U-tests to determine whether there were any significant differences between infected and non-infected individuals for the other assessed infections. Those tests revealed no accuracy differences for CMV (p = .746), HSV (p = .397), EBV (p = .405), *Mycoplasma pneumoniae* (p = .664), Adenovirus (p = .825), or *Chlamydia pneumoniae* (p = .156). RSV and Parainfluenza antibodies were not analyzed as all study participants had been tested positive.

## Materials and Methods

*Assessment of further IgG antibodies*

Chemiluminescence immunoassays were used to detect IgG antibodies against Human Cytomegalovirus (CMV; LIAISON® CMV IgG II, DiaSorin, Saluggia, Italy), Herpes simplex virus 1 and 2 (HSV; LIAISON® HSV-1/2 IgG, DiaSorin,) and Epstein-Barr virus (EBV; LIAISON® VCA IgG and LIAISON® EBNA IgG, both DiaSorin,) in sera of candidates running on a LIAISON® XL (DiaSorin,), a fully automated chemiluminescence analyzer.

IgG antibodies against *Chlamydia pneumoniae* and *Mycoplasma pneumoniae* were detected in sera of candidates using an enzyme immunoassay (SeroCP TM-IgG, Savyon Diagnostics, Ashdod, Israel and Serion ELISA classic Mycoplasma pneumonaie IgG, Virion\Serion GmbH, Würzburg, Germany) running on an ETI-Max 3000 system (DiaSorin, Saluggia, Italy), a semiautomatic enzyme immunoassay analyzer.

In order to determine the IgG antibody concentrations against Respiratory Syncytial virus (RSV), Adenovirus as well as Parainfluenza virus types 1, 2 and 3 the ELISA tests RSV ELISA IgG/IgM, Adenovirus ELISA IgG/IgM and ParaScreen ELISA IgG/IgM (all Sekisui Virotech GmbH, Rüsselsheim, Germany) were used. The tests were performed manually and optical densities were analyzed with a 96-well absorbance microplate reader (Tecan sunrise, Tecan Group Ltd., Männedorf, Switzerland) at 450nm. All parameters were analyzed in duplicates.

*Assessment of Cytomegalovirus IgG antibodies*

Samples with concentrations of anti-CMV-IgG antibodies greater than 14 U/mL were considered reactive for specific IgG antibodies whereas samples with antibody concentrations less than 12 U/mL were considered nonreactive for IgG. Borderline test results have an antibody concentration of ≥ 12 to < 14 U/ml.

*Assessment of Herpes simplex virus 1 and 2 IgG antibodies*

According to the instructions of the manufacturer, samples with anti-HSV 1 and 2-IgG antibodies greater than or equal 1.1 (Index) were considered reactive for specific IgG antibodies. Samples with antibodies less than 0.9 (Index) were considered nonreactive and ≥0.9 to <1.1 (Index) borderline for IgG. Recombinant HSV proteins serve as test antigen.

*Assessment of Epstein-Barr virus IgG antibodies*

Both, IgG antibodies against the viral capsid antigen (VCA) and the Epstein-Barr nuclear antigen (EBNA) of the Epstein-Barr virus were determined.

The p18 synthetic peptide (LIAISON® VCA IgG) and EBNA-1 synthetic peptides (LIAISON® EBNA IgG) serve as antigens.

LIAISON® VCA IgG: samples with concentrations of anti-EBV-VCA-IgG antibodies greater than or equal 20 U/mL were considered reactive for specific IgG antibodies according to the manufacturer´s instructions. Samples with antibody concentrations less than 20 U/mL were considered nonreactive for IgG.

LIAISON® EBNA IgG: according to the manufacturer, samples with anti-EBV EBNA-IgG antibodies greater than 20 U/ml were classified reactive, nonreactive (< 5 U/ml) or borderline (5 to < 20 U/ml).

*Assessment of Chlamydia pneumoniae IgG antibodies*

Cleaned elementary bodies from *Chlamydia pneumoniae* (TWAR 183) serve as test antigen. According to the instructions of the manufacturer, samples with anti-*Chlamydia pneumoniae*-IgG antibodies greater than 1.3 (Index) were considered reactive, nonreactive (< 1.0 Index) or borderline (1.0 to 1.3 Index).

*Assessment of Mycoplasma pneumoniae IgG antibodies*

According to the instructions of the manufacturer, samples with anti-*Mycoplasma pneumoniae*-IgG antibodies greater than 30 IE/ml were considered reactive, nonreactive (< 20 IE/ml) or borderline (20.0 to 30 IE/ml).

*Assessment of Adenovirus IgG antibodies*

Samples with anti-Adenovirus-IgG antibodies greater than 11.0 Virotech units were considered reactive, nonreactive (< 9.0 Virotech units) or borderline (9.0 to 11.0 Virotech units) according to the instructions of the manufacturer.

*Assessment of Parainfluenza IgG antibodies*

The ParaScreen ELISA IgG detects antibodies against the three individual parainfluenza types 1, 2 and 3 in combination. According to the instructions of the manufacturer, samples with anti-Parainfluenza-IgG antibodies greater than 11.0 Virotech units were considered reactive, nonreactive (< 9.0 Virotech units) or borderline (9.0 to 11.0 Virotech units).

*Assessment of Respiratory Syncytial virus (RSV) IgG antibodies*

According to the instructions of the manufacturer, samples with anti-Respiratory Syncytial virus-IgG antibodies greater than 11.0 Virotech units were considered reactive, nonreactive (< 9.0 Virotech units) or borderline (9.0 to 11.0 Virotech units).

## References


1. Henrickson, K. J. Parainfluenza viruses. *Clin. Microbiol. Rev.* **16,** 242–264 (2003).
2. Higgins, C. D. *et al.* A study of risk factors for acquisition of Epstein-Barr virus and its subtypes. *J. Infect. Dis.* **195,** 474–482 (2007).
3. Lübeck, P. R., Doerr, H. W. & Rabenau, H. F. Epidemiology of human cytomegalovirus (HCMV) in an urban region of Germany: what has changed? *Med. Microbiol. Immunol. (Berl.)* **199,** 53–60 (2010).
4. Hellenbrand, W., Thierfelder, W., Müller-Pebody, B., Hamouda, O. & Breuer, T. Seroprevalence of herpes simplex virus type 1 (HSV-1) and type 2 (HSV-2) in former East and West Germany, 1997-1998. *Eur. J. Clin. Microbiol. Infect. Dis. Off. Publ. Eur. Soc. Clin. Microbiol.* **24,** 131–135 (2005).
5. Braun, R. W. & Eggers, M. Respiratory-Sycytial-Virus (RSV). in *Medizinische Virologie. Grundlagen, Diagnostik, Prävention und Therapie viraler Erkrankungen* (eds. Doerr, H. W. & Gerlich, W. H.) 555 (Thieme, 2010).
6. Braun, R. W. & Eggers, M. Parainfluenzaviren. in *Medizinische Virologie. Grundlagen, Diagnostik, Prävention und Therapie viraler Erkrankungen* (eds. Doerr, H. W. & Gerlich, W. H.) 542 (Thieme, 2010).
7. Tuuminen, T. *et al.* Prevalence of Chlamydia pneumoniae and Mycoplasma pneumoniae immunoglobulin G and A antibodies in a healthy Finnish population as analyzed by quantitative enzyme immunoassays. *Clin. Diagn. Lab. Immunol.* **7,** 734–738 (2000).
8. Mast, T. C. *et al.* International epidemiology of human pre-existing adenovirus (Ad) type-5, type-6, type-26 and type-36 neutralizing antibodies: correlates of high Ad5 titers and implications for potential HIV vaccine trials. *Vaccine* **28,** 950–957 (2010).
